# Optimization of the Probiotic Fermentation Process of *Ganoderma lucidum* Juice and Its In Vitro Immune-Enhancing Potential

**DOI:** 10.3390/foods15020227

**Published:** 2026-01-08

**Authors:** Dilireba Shataer, Xin Liu, Yanan Qin, Jing Lu, Haipeng Liu, Liang Wang

**Affiliations:** 1 College of Smart Agriculture (Research Institute), Xinjiang University, Urumqi 830046, China; dlrb_shataer2021@xju.edu.cn (D.S.); l16637462678@163.com (X.L.); qin@xju.edu.cn (Y.Q.); jinglu@xju.edu.cn (J.L.); 2 Centre for Intelligent Healthcare, Coventry University, Coventry CVI 5RW, UK; 3 National Medical Research Association, Leicester, UK; 4 Cardiovascular Analytics Group, Hong Kong SAR, China

**Keywords:** *Ganoderma lucidum* fermented juice (GFJ), mixed fermentation, superoxide dismutase, Ganoderma triterpenoids, immune function

## Abstract

Fermented products have recently garnered substantial interest in both research and commercial contexts. Although probiotic fermentation is predominantly practiced with dairy, fruits, vegetables, and grains, its application to dual-purpose food-medicine materials like *Ganoderma lucidum* has been comparatively underexplored. In this study, *Ganoderma lucidum* fermented juice (GFJ) served as the substrate and was fermented with five probiotic strains. The optimal inoculation ratios—determined by employing a uniform design experiment—were as follows: Bifidobacterium animalis 6.05%, Lacticaseibacillus paracasei 9.52%, Lacticaseibacillus rhamnosus 6.63%, Pediococcus pentosaceus 21.38%, and Pediococcus acidilactici 56.42%. Optimal fermentation parameters established by response surface methodology included 24 h of fermentation at 37 °C, a final cell density of 5 × 10^6^ CFU/mL, and a sugar content of 4.5 °Brix. Experiments with RAW264.7 macrophages revealed that GFJ significantly promoted both phagocytic activity and nitric oxide (NO) secretion, indicating enhanced immune characteristics as a result of fermentation. Untargeted metabolomics profiling of GFJ across different fermentation stages showed upregulation of functional metabolites, including polyphenols, prebiotics, functional oligosaccharides, and Ganoderma triterpenoids (GTs)—notably myricetin-3-O-rhamnoside, luteolin-7-O-glucuronide, raffinose, sesamose, and Ganoderma acids. These increments in metabolic compounds strongly correlate with improved functional properties in GFJ, specifically heightened superoxide dismutase activity and immunomodulatory capacity. These results highlight an effective approach for developing functionally enriched fermented products from medicinal fungi, with promising applications in functional food and nutraceutical industries.

## 1. Introduction

*Ganoderma lucidum* (GL) is a renowned medicinal fungus in Traditional Chinese Medicine (TCM), historically referred to as the “mushroom of immortality” due to its reputed health benefits. Its principal habitats are located in subtropical and tropical regions [1]. China exhibits the highest diversity of GL species, with over 100 distinct variants identified to date [2]. This fungus is a dual-purpose resource for food and medicine and is rich in more than 400 bioactive compounds, including triterpenoids, polysaccharides, and proteins, all known for diverse pharmacological activities such as antioxidation, antitumor, anti-inflammation, hepatoprotection, and immunomodulatory properties [3]. Products developed from GL commonly include spore powder, medicinal wines, ointments, and teas, reflecting its versatility and importance in both dietary and pharmaceutical applications [4,5].

Recently, the market for fermented products has expanded markedly, primarily through the transformation of raw materials into higher-value functional products by means of probiotic fermentation [6]. The fermentation of lactic acid bacteria promotes the extraction of active compounds from Traditional Chinese Medicine and enhances both sensory and nutritional traits of foods [7]. Additionally, fermentation facilitates the conversion of primary metabolites into more bioactive forms—such as generating aglycones from glycosides—mitigates the bitterness associated with Ganoderma triterpenoids, and results in the production of beneficial postbiotics (e.g., organic acids, bacteriocins), thereby collectively augmenting substrate functionality [8]. To date, probiotic fermentation has mainly been exploited for dairy, fruit, vegetable, and grain substrates [9], while research involving dual-purpose medicinal-edible fungi like GL remains scarce [10]. Notably, fermentation can improve the antioxidant capacity of spore powder polysaccharides [11]. Solid-state fermentation of GL can enhance both grain quality and functional properties, suggesting promising roles for fermented fungi in functional food and medicine [12]. However, systematic investigation of GL fermentation, especially using mixed cultures of lactic acid bacteria (LAB), is limited. Mixed-culture fermentation systems take advantage of microbial synergy, potentially increasing the bioconversion of bioactives, improving flavor, and providing greater process stability compared to single-strain fermentation approaches [13]. To address the current gap in research, the present study selected five LAB strains for mixed-culture fermentation of GL fruit juice. The inoculation ratio was optimized using a uniform design experiment, while process conditions were refined using response surface methodology, with superoxide dismutase (SOD) activity and Ganoderma triterpenoids (GTs) content used as primary evaluation indexes. The immunomodulatory properties of *Ganoderma lucidum* fermented juice (GFJ) were subsequently assessed at the cellular level. Finally, untargeted metabolomics was deployed to comprehensively analyze metabolite variations across fermentation stages. Comparative analysis of bioactive compounds confirmed that fermentation enhances the bioactivity profile of GFJ, providing useful insights for the development of functional foods and pharmaceuticals derived from GL.

## 2. Materials and Methods

GL fruiting bodies were sourced from Changzhou, Jiangsu Province, China. The following microbial strains were obtained from the China Center for Type Culture Collection (CCTCC, Beijing, China): *Pediococcus acidilactici*, *Pediococcus pentosaceus*, *Lacticaseibacillus paracasei*, *Lacticaseibacillus rhamnosus*, *Bifidobacterium animalis* subsp. *animalis*, *Lactiplantibacillus plantarum*, *Lactobacillus acidophilus*, *Limosilactobacillus fermentum*, and *Streptococcus thermophilus*. Enzymes, including pectinase (500 U/mg), cellulase (50 U/mg), and xylanase (20 U/mg) were purchased from Shanghai Yuanye Bio-Technology Co., Ltd. (Shanghai, China). The murine monocyte-macrophage cell line RAW264.7 was procured from Procell Life Science & Technology Co., Ltd. (Wuhan, China). Assay kits for SOD (superoxide dismutase) activity were provided by Nanjing Jiancheng Bioengineering Institute (Nanjing, China).

### 2.1. Bacterial Strain Activation

LAB strains were cultivated in MRS liquid medium at 37 °C under anaerobic conditions for 24 h in a thermostatic incubator, and subsequently subcultured 2–3 times prior to use in fermentation experiments.

### 2.2. Sample Preparation

GL fruiting bodies were rinsed with distilled water to eliminate surface debris and dust. The cleaned material was combined with distilled water at a ratio of 1:15 (*w*/*v*) and homogenized via mechanical blending. The resulting slurry was subjected to microwave pretreatment (750 W, 40 s) followed by ultrasonication (730 W, 15 min). The total soluble solids (TSS) were adjusted to 4.5 °Brix. Enzymatic hydrolysis was conducted with 0.2% (*w*/*v*) pectinase, 0.1% (*w*/*v*) cellulase, and 0.1% (*w*/*v*) xylanase at 55 °C for 3 h. The sample was then sterilized at 90 °C for 20 min before subsequent use in fermentation.

### 2.3. Selection of the Fermentation Strains and Determination of Their Proportions

GL juice was fermented using nine bacterial strains at an inoculum density of 5 × 10^6^ CFU/mL and TSS of 4.5 °Brix at 37 °C. The selection of optimal strains was based on measured SOD (superoxide dismutase) activity, Ganoderma triterpenoids (GTs) content, pH, sensory evaluation, and endpoint attainment time. Optimization was performed using a uniform design table U105 (Table S1). SOD activity served as the response variable; quadratic polynomial stepwise regression analysis using SPSS 26.0 software generated the fermentation model, with maximum SOD activity as the objective. Solver functions in Excel were used to determine the optimal inoculation percentages for the five key strains.

### 2.4. Optimization of the GFJ Fermentation Process Using Response Surface Methodology (RSM)

Upon finalizing the strains and their ratios, SOD activity and GTs content post-fermentation were the main indicators for further optimization. Single-factor testing for GFJ production involved varying fermentation time (20, 24, 28, 32, 36 h), temperature (27, 32, 37, 42, and 47 °C), inoculum volume (2, 5, 8, 11, and 14 × 10^6^ CFU/mL), and total soluble solids (3.0, 3.5, 4.0, 4.5, and 5.0 °Brix). Based on results from the single-factor experiments, further optimization was performed using response surface methodology (RSM) with a Box–Behnken design [14]. The design parameters and factor levels are detailed in Table S2, facilitating the production of higher-quality GFJ.

### 2.5. Physicochemical Index Analysis

#### 2.5.1. pH Measurement

The pH values of the samples were determined using a digital benchtop pH meter (Model PHS-3C, Shanghai, China), which was calibrated against standard buffer solutions at pH 4.0 and 6.8 prior to each assay.

#### 2.5.2. Total Soluble Solids (TSS) Content

The total soluble solids (TSS) content was quantified using a digital refractometer (Model TD-45, Hangzhou, China). After dispensing 1 mL of the sample onto the prism surface, the °Brix value was recorded following automatic temperature compensation.

#### 2.5.3. SOD Activity

The activity of SOD (superoxide dismutase) was assessed according to the manufacturer’s instructions provided with the SOD assay kit (Nanjing Jiancheng Bioengineering Institute, Nanjing, China).

#### 2.5.4. GTs Content

The Ganoderma triterpenoids (GTs) content was measured following the method described by references therein [15]. For sample preparation, 10 mg of GL juice was extracted in 10 mL of ethyl acetate. The extract was evaporated to dryness in a water bath at 100 °C. The residue was combined with 0.4 mL of 5% vanillin-glacial acetic acid and 1.0 mL perchloric acid, heated at 60 °C for 15 min, and then cooled immediately in an ice-water bath. Subsequently, 5 mL of glacial acetic acid was added and thoroughly mixed. Absorbance was measured at 548 nm using a UV-Vis spectrophotometer.

#### 2.5.5. Sensory Evaluation

Sensory attributes of GFJ were evaluated using a standardized methodology as described in previous reports [16]. Panelists were trained according to ISO guidelines to ensure consistency and accuracy in sensory evaluation. Detailed sensory descriptors and evaluation criteria are presented in Table S3, allowing for comprehensive characterization of GFJ’s sensory profile.

### 2.6. Cell Experiments

#### 2.6.1. Cell Culture

RAW264.7 macrophages were thawed, passaged, and stored according to the procedures described by Wujunhua and colleagues [17].

#### 2.6.2. MTT Assay for Cell Viability Determination

To assess the impact of fermentation on immunomodulatory properties, both unfermented GL juice (control group) and GFJ were evaluated. An LPS (lipopolysaccharide)-stimulated RAW264.7 macrophage model was constructed, and cell viability was measured by MTT assay. RAW264.7 cells were seeded in 96-well plates and incubated for 24 h. Following removal of the supernatant, cells were exposed to various concentrations of GFJ for an additional 24 h. Each well then received 100 µL of MTT solution (0.5 mg/mL), followed by a 3–4 h incubation. After centrifugation and removal of supernatant, formazan crystals were dissolved in DMSO (150 µL/well). Absorbance was recorded at 490 nm. Cell viability was calculated using the standard MTT method.

#### 2.6.3. Effect of GFJ on Phagocytic Activity of RAW264.7 Cells

RAW264.7 cells were seeded in 96-well plates and cultured for 24 h. After removing the supernatant, cells were treated with 100 µL GFJ (0.1–0.5 mg/mL) or LPS (1 µg/mL; model group) for 24 h. The medium was replaced with 0.075% neutral red for 4 h. Post-washing with PBS, 150 µL lysis buffer (glacial acetic acid:ethanol = 1:1) was added and incubated for another 4 h. Absorbance was measured at 540 nm, and phagocytic rate was calculated accordingly.

#### 2.6.4. Effect of Different Concentrations of GFJ on NO Secretion in RAW264.7 Cells

After processing cells as described in Section 2.6.3, the supernatant from RAW264.7 cells was collected and analyzed using the Nanjing Jiancheng NO assay kit as per the manufacturer’s instructions.

#### 2.6.5. Fluorescence Inverted Microscope Observation

RAW264.7 cells were seeded into 96-well plates and incubated for 24 h. After treatment with LPS (1 µg/mL) and varied GFJ concentrations for an additional 24 h, cells were washed, fixed using 4% formaldehyde, and stained with DAPI in darkness. Images were taken with a fluorescence microscope after PBS washing.

### 2.7. LC-MS/MS Analysis

Metabolite analysis was performed using two LC/MS methods for all samples. One aliquot was analyzed under positive ion mode on a Waters ACQUITY Premier HSS T3 column (1.8 µm, 2.1 mm × 100 mm), eluted with 0.1% formic acid in water (solvent A) and 0.1% formic acid in acetonitrile (solvent B) using a gradient: 5–20% in 2 min, to 60% over 3 min, ramped to 99% in 1 min, held 1.5 min, returned to 5% in 0.1 min, held for 2.4 min. Conditions: column temperature 40 °C; flow rate 0.4 mL/min; injection volume 4 µL. The second aliquot, analyzed in negative ion mode, used the same elution gradient. Data acquisition utilized information-dependent acquisition (IDA) mode with Analyst TF 1.7.1 Software (Sciex, Framingham, MA, USA). Source settings included ion source gas 1/2 (50 psi), curtain gas (25 psi), temperature (550 °C), declustering potential (60 V in positive, −60 V in negative mode), and ion spray voltage floating (5000 V positive, −4000 V negative). TOF MS scans were performed over 50–1000 Da (200 ms accumulation, dynamic background subtraction ‘on’). Product ion scans: 25–1000 Da (40 ms), collision energy (30 V positive, −30 V negative), spread 15, resolution UNIT, charge state 1, intensity 100 cps, isotope exclusion within 4 Da, mass tolerance 50 ppm, max 18 candidate ions per cycle.

### 2.8. Statistical Analysis

Untargeted metabolomics analysis employed six parallel samples, while all other measurements utilized triplicate samples. Results are presented as mean ± standard deviation. Data analysis utilized SPSS 26.0 (SPSS Science, Chicago, IL, USA). One-way analysis of variance (ANOVA) with Duncan’s multiple comparisons test (or non-parametric test when the preconditions are violated) was applied, considering *p* < 0.05 statistically significant. Design-Expert13 software was used for response surface and contour mapping. The ‘anal’ function in the MetaboAnalystR package (version 1.0.1.) facilitated metabolomic analysis, while the ‘prcomp’ function in R performed principal component analysis (PCA). Differential metabolites were identified based on criteria VIP ≥ 1, *p* < 0.05, and fold change (FC) ≥ 2 or ≤0.5. Visualizations, including line charts, bar graphs, and heatmaps, were generated in Origin 2024 (OriginLab Corporation, Northampton, MA, USA).

## 3. Results and Discussion

### 3.1. Strain Screening

Compared to single-strain fermentation, mixed-strain fermentation offers significant advantages. Specifically, multi-strain lactic acid bacteria co-fermentation systems exhibit superior growth kinetics through interspecies interactions and synergistically enhance metabolic products [18]. In recent years, research on mixed-strain fermentation has garnered increasing attention. Research by Li et al. indicates that mixed fermentation of quinoa by diverse lactic acid bacteria strains substantially increases free phenolic content and antioxidant activity [19]. Furthermore, mixed-strain fermentation effectively elevates flavor-enhancing volatile compounds while reducing the formation of undesirable flavor compounds [7]. Therefore, we first conducted fermentation strain screening. The results are shown in Table 1. Testing revealed that all nine lactic acid bacteria strains exhibited varying degrees of enhanced superoxide dismutase (SOD) activity after fermentation, with Lactobacillus rhamnosus demonstrating the most significant improvement. As an enzyme highly effective at scavenging free radicals [20], SOD activity can be further enhanced through lactic acid bacteria metabolism [21]. Although GL naturally contains SOD, lactic acid bacteria metabolism significantly enhances its activity. GL is rich in Ganoderma triterpenoids [22,23]. All nine strains (GTs) confer substantial pharmacological effects, with Lactobacillus paracasei exhibiting the highest GT concentration after fermentation. pH measurements revealed differences in acid production capacity among strains, with Lactobacillus pentosus demonstrating the strongest acid-producing ability. When developing novel foods, nutritional and functional properties are paramount, yet sensory characteristics remain crucial. Streptococcus thermophilus, Lactobacillus plantarum, Lactobacillus fermentum, and Lactobacillus acidophilus received low sensory scores, indicating unacceptable flavor and texture, thus eliminating these four strains. Fermentation duration is equally critical in industrial production, as extended cycles increase costs. Lactobacillus pentosus exhibited the shortest fermentation cycle. After comprehensive evaluation of superoxide dismutase activity, fructosyl glycoside content, acid production capacity, sensory evaluation, and fermentation time across nine lactic acid bacterial strains, the final fermentation strains selected were: Lactobacillus rhamnosus, Lactobacillus paracasei, Lactobacillus pentosus, Lactobacillus acidophilus, and Bifidobacterium animalis.

### 3.2. GFJ Fermentation Process Optimization

Based on the screening results, a uniform design experiment was performed, detailed in Table S4. The final regression model was established and evaluated as follows: The regression equation demonstrated strong reliability (R^2^ = 0.995, *p* < 0.05), supporting its predictive accuracy for the optimal mixed-culture inoculation ratio. The predicted ratio was: Bifidobacterium animalis 6.05%, Lacticaseibacillus paracasei 9.52%, Lacticaseibacillus rhamnosus 6.63%, Pediococcus pentosaceus 21.38%, and Pediococcus acidilactici 56.42%, with an anticipated SOD activity of 123.45 U/g. Validation experiments yielded SOD activity of 122.51 U/g, closely matching model predictions. Relative to the maximal SOD activity from single-strain fermentation (121.67 U/g), the mixed-culture system delivered superior outcomes.

### 3.3. Response Surface Experimental Results

**Figure 1:**
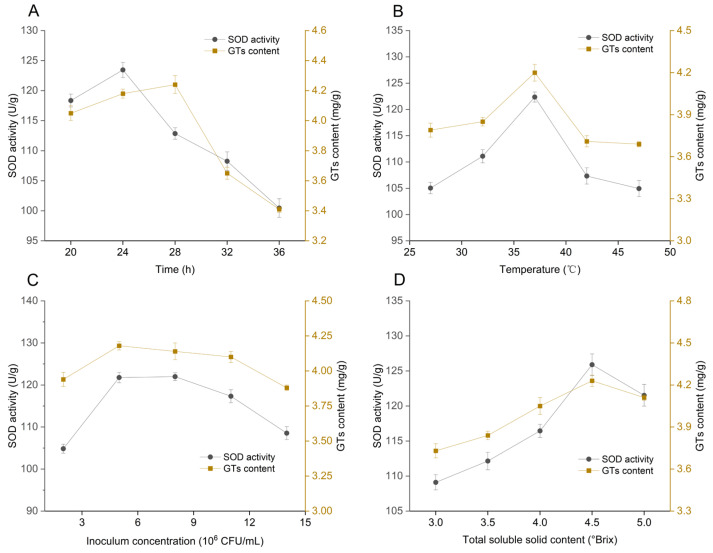
(**A**) The effect of fermentation time on GFJ; (**B**) The effect of temperature on GFJ; (**C**) The effect of inoculation amount on GFJ; (**D**) Effect of total soluble solids content on GFJ.

As depicted in Figure 1A–D, optimal results were obtained with fermentation at 37 °C for 24 h, using an inoculation density of 5 × 10^6^ CFU/mL and a TSS of 4.5 °Brix. Under these conditions, SOD activity and GTs content reached peak levels (125.89 U/g and 4.23 mg/g, respectively), representing an improvement over the best single-strain fermentation (Lacticaseibacillus rhamnosus: 121.67 U/g SOD, 3.46 mg/g GTs). This corresponds to 3.9% and 26.6% respective increases, providing clear evidence of the advantages of optimized mixed-culture fermentation. Declines in SOD activity and GTs content beyond the optimal fermentation time and temperature likely result from excessive fermentation, acid build-up, and inhibited microbial and enzymatic activity [24,25]. Metabolomics results (Section 3.5 indicated a pronounced down-regulation of metabolites after 18 h, mirroring this decline. Elevated inoculation levels or temperatures further destabilized metabolites and reduced enzymatic activities [26]. Accordingly, fermentation time (20 h, 24 h, 28 h), temperature (32 °C, 37 °C, 42 °C), inoculation concentration (2 × 10^6^ CFU/mL, 6 × 10^6^ CFU/mL, 8 × 10^6^ CFU/mL), and TSS (4, 4.5, 5) were selected for subsequent Box–Behnken design experiments.

**Figure 2:**
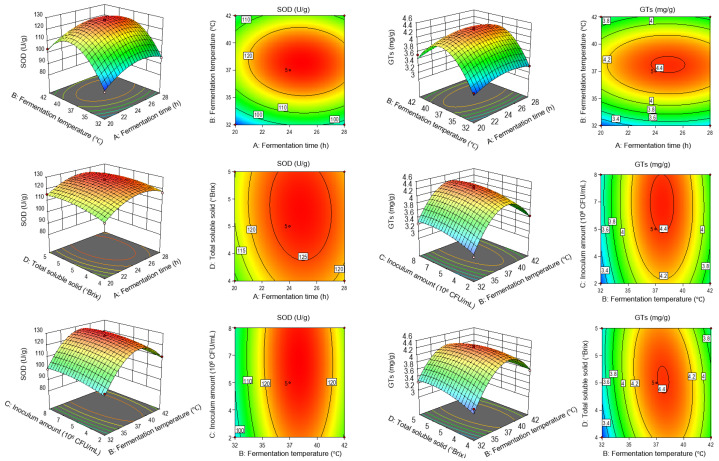
Effects of interaction of various factors on SOD activity and GTs of GFJ.

Based on single-factor results and the Box–Behnken central composite design methodology, fermentation time (A), temperature (B), inoculation concentration (C), and TSS (D) were chosen as independent variables, while SOD activity (Y1) and GTs content (Y2) served as response variables. The results of the response surface test are presented in Table S5. Analysis of variance (ANOVA) and significance testing (Table S6) indicate both regression models are highly significant (*p* < 0.0001) with a non-significant lack of fit. High R^2^ values (0.9880 for SOD activity, 0.9581 for GTs content) support the models’ predictability. Relative factor impacts, based on F values, were ranked for SOD activity: fermentation temperature (B) > fermentation time (A) > sugar addition (D) > inoculum concentration (C). For GTs content: fermentation temperature (B) > inoculum concentration (C) > fermentation time (A) > sugar addition (D). Response surface and contour analyses (Figure 2) depict the interactions among optimization variables. Downward-opening surfaces confirm response values (SOD and GTs) initially increase to maxima before declining. Elliptical contours denote statistically significant interactions. The model predicted optimal fermentation conditions as: 24.83 h, 37.56 °C, 5.43 × 10^6^ CFU/mL inoculum, and 4.53 °Brix, resulting in expected SOD activity of 128.25 U/g and GTs content of 4.44 mg/g. To facilitate real-world application, conditions were refined to 24 h, 37 °C, 5 × 10^6^ CFU/mL, and 4.5 °Brix. Experimentally, these yielded SOD activity of 126.45 U/g and GTs content of 4.38 mg/g, with relative errors of 1.40% and 1.35%, validating the process model.

### 3.4. The Immunomodulatory Properties of GFJ

**Figure 3:**
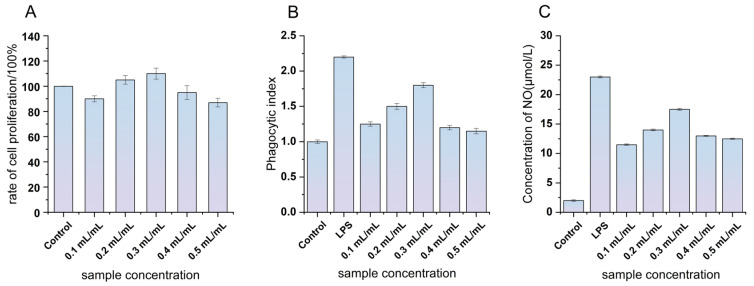
Effects of different concentrations of GFJ on RAW264.7 cells (**A**): cell proliferation rate. (**B**): phagocytic activity. (**C**): NO level secreted by cells.

As depicted in Figure 3A, GFJ at different concentrations influenced RAW264.7 survival rates over 24 h (*p* < 0.05). Compared to a baseline control survival rate of 100%, GFJ concentrations first enhanced, then reduced, cell survival. Notably, proliferation rates exceeded 100% at concentrations of 0.2–0.4 mL/mL, indicating that GFJ was non-cytotoxic within this range and could promote cell growth.

#### 3.4.1. Effect of GFJ Concentration on the Phagocytic Activity of RAW264.7 Cells

Neutral red, a large molecular weight fluorescent dye with a notable absorption peak at 550 nm, is selectively internalized by macrophages via endocytosis [27]. Thus, quantification of intracellular neutral red provides a robust indicator of macrophage phagocytic activity under various treatments. In Figure 3B, assuming a baseline phagocytic index of 1 for the control group, GFJ treatment resulted in an initial increase, then a decrease, in RAW264.7 cell phagocytic capacity across concentrations. The LPS group, as well as 0.1, 0.2, and 0.3 mL/mL GFJ groups, all showed significant enhancement of phagocytosis. A peak phagocytic index (1.80 ± 0.16) was observed at 0.3 mL/mL, suggesting that GFJ at this concentration robustly activates macrophages and thereby strengthens immunomodulatory capacity. Previous studies have shown similar activation with GL extract (e.g., rFIP-glu) at 2 µg/mL in RAW264.7 macrophages, further supporting the improvement of GFJ immunity [28].

#### 3.4.2. Effect of GFJ on NO Secretion in RAW264.7 Cells

Nitric oxide (NO) is a critical signaling molecule, widely present in both intra- and extracellular environments, and plays important roles in multiple physiological and pathological processes, including immune regulation [29]. As one of the principal effector products of macrophages, NO enhances cellular activity and mediates both innate and adaptive immune responses [30]. As indicated in Figure 3C, GFJ significantly increased NO secretion at all tested concentrations compared to the control group (*p* < 0.05). Further, at 0.2–0.4 mL/mL, NO levels were significantly higher than in the LPS-stimulated group, underscoring GFJ’s capacity to boost cellular immune responses. This finding aligns with reports that Ganoderma polysaccharide-peptide—comprising polysaccharides from GL and Polyporus umbellatus—also increased NO release in RAW264.7 macrophages [31].

#### 3.4.3. Fluorescence Inversion Microscopy Analysis

DAPI dye enables simultaneous staining of live and fixed cells, providing visual enumeration of macrophages and indirect assessment of non-specific immune enhancement [32]. As illustrated in Figure S1, cell counts in both GFJ-treated and LPS-stimulated groups differed markedly from the control. Increasing GFJ concentration from 0.2 to 0.4 mL/mL led to a statistically significant rise in cell numbers, while concentrations above 0.4 mL/mL resulted in decreased counts. At 0.3 mL/mL, cell numbers approached those detected in the LPS control group. In summary, both unfermented GL juice and GFJ enhanced phagocytic activity and NO secretion in RAW264.7 macrophages, with GFJ consistently exerting a greater effect at equivalent concentrations. Maximal immunomodulatory benefit was observed at 0.3 mL/mL, indicating that fermentation significantly potentiates the cellular immune function of GL juice.

### 3.5. Untargeted Metabolomics Analysis

**Figure 4:**
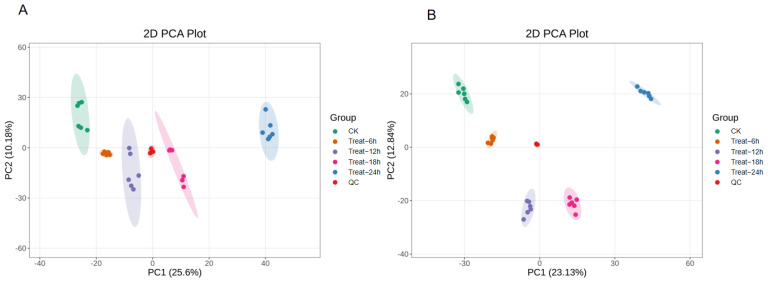
PCA in positive ion (**A**) and negative ion (**B**) modes.

To elucidate the metabolic basis underlying the enhanced functionality of GFJ observed under optimized fermentation conditions (see Section 3.2 and Section 3.3), an untargeted metabolomics approach was utilized, identifying a total of 4030 metabolites across fermentation stages—2382 in positive ion mode and 1648 in negative ion mode. Principal component analysis (PCA; Figure 4) revealed that replicate samples within each group clustered tightly, confirming high within-group similarity, whereas clear separation between groups indicated substantial metabolite shifts during fermentation [33]. Quality control (QC) samples clustered centrally within the PCA plot, attesting to data reliability and model stability.

#### 3.5.1. Screening of Differential Metabolites

**Figure 5:**
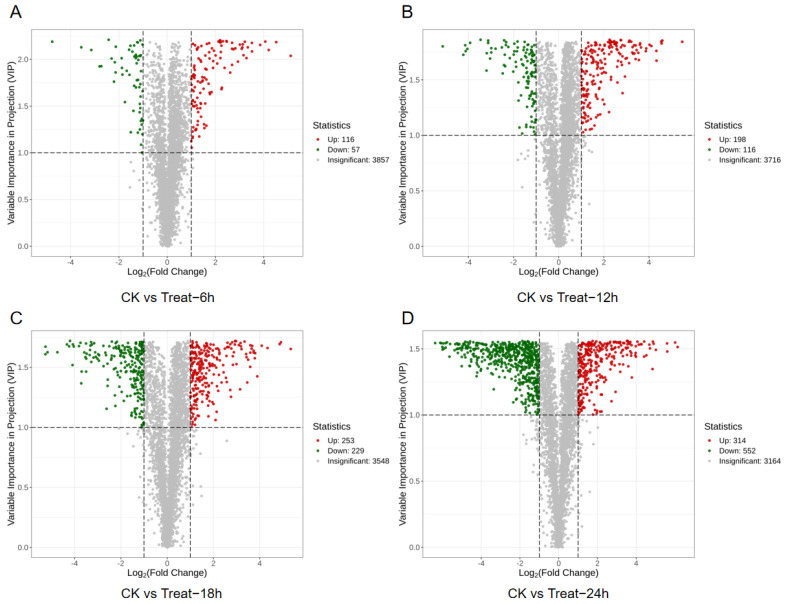
The differential metabolite volcano plot: (**A**): CK vs. 6 h; (**B**) CK vs. 12 h; (**C**) CK vs. 18 h; (**D**) CK vs. 24 h (with red dots representing upregulated metabolites, green dots indicating downregulated metabolites, and gray dots denoting metabolites without significant differences).

A total of 1584 differential metabolites were found among the four experimental groups. Visualizations using volcano plots (Figure 5) intuitively depict temporal changes in metabolite profiles between unfermented and various fermentation stages [34]. The number of differential metabolites increased progressively during fermentation; up-regulated compounds predominated early, indicating intensified metabolic activity [35]. However, as fermentation advanced and nutrients were depleted, pH decreased and metabolic activity waned, leading to marked increases in down-regulated metabolites after 18 h [36]. This is consistent with the phenomenon found in the previous optimization of fermentation time.

#### 3.5.2. Classification of Differential Metabolites

**Figure 6:**
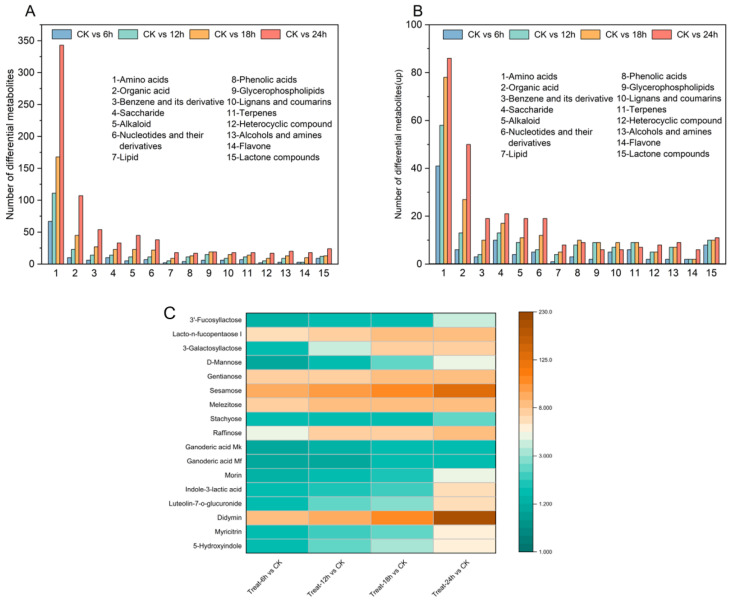
(**A**). The number of differential metabolites in different stages of GFJ fermentation; (**B**). The number of up-regulated metabolites at different stages of GFJ fermentation; (**C**). The varying fold increases in content of the 17 upregulated metabolites across different fermentation stages compared to the unfermented group.

Major categories of differentially expressed metabolites during GFJ fermentation were assigned based on VIP values. Detected species included amino acids and derivatives, organic acids, aromatic compounds, sugars, alkaloids, nucleotides, lipids, phenolic acids, glycerophospholipids, lignans, coumarins, terpenoids, heterocycles, alcohols, amines, flavonoids, lactones, and more (Figure 6A). Although total differential metabolites increased with fermentation time, the proportion of down-regulated metabolites rose, while up-regulated metabolites, such as phenolic acids, glycerophospholipids, terpenoids, lignans, and coumarins, decreased after 18 h (Figure 6B). This pattern, observed previously in black mulberry juice fermentation, underscores the importance of optimal fermentation duration. Up-regulated compounds included several bioactive substances—particularly flavonoids, terpenoids, prebiotics, and functional oligosaccharides—demonstrating that fermentation markedly enhances GFJ quality [37].

#### 3.5.3. The Relationship Between Differential Metabolites and Functional Activity

The up-regulation of functional metabolites—such as flavonoids, terpenoids, and prebiotics—appears closely related to improvements in SOD activity and immunomodulatory properties observed in GFJ (Figure 6C). For example, elevated concentrations of flavonoids (myricetin-3-O-rhamnoside and luteolin-7-O-glucuronide) may contribute to greater SOD activity by activating the Nrf2 antioxidant pathway, which modulates antioxidant enzyme expression, including SOD [38,39,40]. Other notable antioxidants included 5-hydroxyindole and indole-3-lactic acid; the latter, produced by lactic acid bacteria from tryptophan metabolism, is recognized as a potent antioxidant and anti-inflammatory compound that supports intestinal barrier integrity and immune regulation via the Ah receptor pathway—beneficial for both gut and neurological health [41]. These polyphenols and antioxidants collectively enhance SOD activity by direct and indirect mechanisms. Notably, ganoderic acid Mk and Mf, important tetracyclic triterpenoids in GL, are known for their immunomodulatory and anti-inflammatory effects [42]; increased abundance following fermentation signals improvement in GTs content in GFJ. The immunomodulatory effects of prebiotics such as raffinose and functional oligosaccharides can be attributed to their remodeling of the gut microbiome and/or direct interactions with immune cells via non-microbiome-dependent mechanisms [43]. D-mannose is a naturally occurring monosaccharide known to enhance immune system regulation and play a crucial role in innate immune defense. Elevated concentration may positively influence the immunological characteristics of GFJ [44]. Additionally, GFJ exhibits elevated levels of 3′-galactosylgalactose, 3′-fucosylgalactose, and lactose—compounds structurally analogous to the complex oligosaccharides found in human milk oligosaccharides (HMOs). While HMOs themselves lack nutritional value, they provide seeds for beneficial gut microbiota, directly promoting bifidobacteria colonization [45]. Simultaneously, they resist pathogen adhesion and actively modulate immune development—a property crucial for maintaining a healthy gut microbiota and systemic immunity. While strong correlations between metabolite up-regulation and functional improvement have been observed, more targeted studies (e.g., pathway inhibition assays or isolation of specific bioactives) are needed to confirm direct causality.

This study optimized the fermentation process of GFJ using a mixture of five lactic acid bacteria through uniform design and response surface methodology, significantly enhancing its SOD activity and triterpenoid content. Cell experiments confirmed that GFJ enhances macrophage phagocytic function and NO secretion, indicating its immunomodulatory potential. Metabolomics further revealed that fermentation promoted the accumulation of bioactive compounds such as flavonoids, ganoderic acids, and functional oligosaccharides, providing a material basis for its functional enhancement. However, limitations remain: the selected strain combinations and fixed process parameters may restrict the generalizability of conclusions; laboratory-scale studies did not address pilot-scale upscaling and production consistency challenges; non-targeted metabolomics may overlook low-abundance metabolites and microbial interactions. Future research should expand to more strain combinations, validate findings through pilot-scale and animal studies, and employ targeted metabolomics and molecular experiments to clarify causal links between key bioactive components and immune functions. This study provides a valuable foundation for developing functional fermented *Ganoderma lucidum* beverages.

## 4. Conclusions

Fermentation of GL juice with a selected mixture of lactic acid bacteria efficiently improved its in vitro bioactivity and immunomodulatory potential. The process facilitated bioconversion of active compounds, including polyphenols, Ganoderma triterpenoids, and prebiotics, which collectively were associated with enhanced antioxidant and immune-enhancing effects in cellular models. These results highlight an effective approach for developing functionally enriched fermented products from medicinal fungi, with promising applications in functional food and nutraceutical industries. However, further research, including in vivo animal studies and clinical trials, is necessary to substantiate these health-promoting effects.

## Figures and Tables

**Table 1:** Physicochemical properties of GFJ fermented by nine strains.

NO.	Strains	SOD Activity (U/g)	GTs (mg/g)	pH	Sensory Evaluation (Score)	Fermentation End Time (h)
1	*G. lucidum* juice	95.26 ± 2.67 ^g^	3.02 ± 0.01 ^d^	4.27 ± 0.02 ^a^	83.89 ± 1.13 ^b^	/
2	*B. animalis*	110.00 ± 2.00 ^d^	3.42 ± 0.01 ^ab^	3.46 ± 0.03 ^b^	88.33 ± 2.08 ^a^	24.0 ± 0.00 ^e^
3	*L. paracasei*	117.33 ± 1.16 ^b^	3.48 ± 0.01 ^a^	3.38 ± 0.05 ^c^	84.33 ± 2.08 ^b^	25.0 ± 0.50 ^d^
4	*P. pentosaceus*	105.67 ± 2.08 ^ef^	3.46 ± 0.01 ^a^	3.20 ± 0.08 ^g^	76.33 ± 1.53 ^c^	23.5 ± 0.50 ^e^
5	*L. rhamnosus*	121.67 ± 1.53 ^a^	3.46 ± 0.01 ^a^	3.23 ± 0.01 ^f^	81.33 ± 1.53 ^b^	27.1 ± 0.29 ^c^
6	*S. thermophilus*	114.33 ± 2.52 ^c^	3.44 ± 0.01 ^ab^	3.29 ± 0.03 ^d^	67.00 ± 1.00 ^d^	24.5 ± 0.50 ^b^
7	*L. plantarum*	102.33 ± 1.53 ^f^	3.42 ± 0.01 ^ab^	3.26 ± 0.04 ^e^	65.00 ± 1.00 ^d^	36.5 ± 0.00 ^a^
8	*P. acidilactici*	110.33 ± 2.08 ^d^	3.38 ± 0.02 ^bc^	3.35 ± 0.07 ^c^	76.33 ± 2.08 ^c^	24.0 ± 0.50 ^c^
9	*L. fermentum*	106.00 ± 1.00 ^e^	3.33 ± 0.10 ^c^	3.30 ± 0.03 ^d^	67.67 ± 1.53 ^d^	24.0 ± 0.29 ^d^
10	*L. acidophilus*	103.00 ± 1.26 ^f^	3.28 ± 0.01 ^c^	3.36 ± 0.02 ^c^	67.46 ± 1.65 ^d^	25.0 ± 0.00 ^d^

Results are expressed as mean values ± SD. Distinct letters adjacent to data in the same column indicate statistically significant differences (*p* < 0.05).

## Data Availability

The original contributions presented in this study are included in the article/Supplementary Material. Further inquiries can be directed to the corresponding authors.

## Supplementary Materials

The following supporting information can be downloaded at: https://www.mdpi.com/article/10.3390/foods15020227/s1, Table S1: Uniform design table of the experiment; Table S2: Experimental factor level table; Table S3: Sensory attributes for *Ganoderma lucidum* fermented juice; Table S4: Results of uniform design experiments; Table S5: Response surface optimization results of GFJ; Table S6: Analysis of variance of SOD activity and GTs in GFJ; Figure S1: fluorescence inverted microscope observation: A: control group; B: LPS group; C: 0.1 mL/mL; D: 0.2 mL/mL; E: 0.3 mL/mL; F: 0.4 mL/mL; G: 0.5 mL/mL.
