# Supplementary material for: Optimization of the Probiotic Fermentation Process of Ganoderma lucidum Juice and Its In Vitro Immune-Enhancing Potential
Source: Foods. 2026 Jan 8;15(2):227. doi: 10.3390/foods15020227 (PMC12839947; doi:10.3390/foods15020227)
Supplement: Supplementary file 1 [file foods-15-00227-s001.zip › foods-4030903-supplementary.pdf]

Table S1. Uniform design table of the experiment

NO.	*B.animalis* X_1/%	*L.paracasei* X_2 /%	*L. rhamnosus* X_3%	*S.thermophilus* X_4/%	*P.acidilactici* X_5 /%
1	0.20	0.10	0.30	0.20	0.10
2	0.25	0.20	0.40	0.30	0.15
3	0.30	0.30	0.50	0.40	0.20
4	0.35	0.40	0.60	0.50	0.25
5	0.40	0.50	0.70	0.60	0.30
6	0.45	0.60	0.80	0.70	0.35
7	0.50	0.70	0.90	0.80	0.40
8	0.55	0.80	1.00	0.90	0.45
9	0.60	0.90	1.10	1.00	0.50
10	0.65	1.00	1.20	1.10	0.55

Table S2. Experimental factor level table.

Factors	Code	Levels		
		−1	0	1
Fermentation time (h)	A	20	24	28
Fermentation temperature (°C)	B	32	37	42
Inoculum concentration (10^6 CFU/mL)	C	2	5	8
Soluble solid content (°Brix)	D	4	4.5	5

Table S3. Sensory attributes for *Ganoderma lucidum* fermented juice.

Category	Score criteria			
Tincture	Even color, amber or light brown. (20-16)	The color is uniform, slightly darker or lighter. (15-11)	The color is basically normal, but there is a small amount of precipitation. (6-10)	The color is dim, with obvious precipitation. (1-5)

fragrance	The coordination of Ganoderma lucidum medicinal aroma and lactic acid bacteria fermentation aroma (20-16)	*Ganoderma lucidum* or fermented fragrance slightly prominent (15-11)	Aroma is light, or slightly rancid. (6-10)	The aroma is not harmonious, with obvious pungent and sour taste. (1-5)
taste	The sweet and sour is moderate, the bitter taste of Ganoderma lucidum is soft. (20-16)	The sweet and sour is slightly stronger or weaker, and the bitter taste is acceptable (15-11)	The sour or bitter taste is more prominent. (6-10)	Excessively sour, bitter or astringent taste is obvious. (1-5)
tasted	Smooth taste, uniform texture. (20-16)	Slight graininess or viscosity (15-11)	There is obvious precipitation or slight roughness. (6-10)	Sticky taste or too thin, swallowing discomfort (1-5)
acceptable degree	Strongly like (20-16)	Moderately like (15-11)	Neutral (6-10)	Dislike (1-5)

Table S4. Results of uniform design experiments.

NO.	*B.animalis* X_1/%	*L.paracaSei* X_2 /%	*L.rhamnosus* X_3%	*S.thermophilus* X_4/%	*P.acidilactici* X_5 /%	SOD U/g
1	0.20	0.10	0.30	0.20	0.10	106.30±0.66[d]
2	0.25	0.20	0.40	0.30	0.15	110.12±0.85[c]
3	0.30	0.30	0.50	0.40	0.20	116.85±0.84[a]
4	0.35	0.40	0.60	0.50	0.25	100.14±0.56[e]
5	0.40	0.50	0.70	0.60	0.30	100.79±0.57[e]
6	0.45	0.60	0.80	0.70	0.35	115.38±0.44[a]
7	0.50	0.70	0.90	0.80	0.40	115.52±0.97[a]
8	0.55	0.80	1.00	0.90	0.45	87.61±1.07f
9	0.60	0.90	1.10	1.00	0.50	110.47±0.77c

10	0.65	1.00	1.20	1.10	0.55	111.83±0.54^{b}

Note: Data are expressed as means ± SDs. Different letters next to data in the same column indicate significant differences between data ($p < 0.05$).

Table S5 Response surface optimization results of GFJ

**Run**	**Fermentation time (h)**	**Fermentation temperature (°C)**	**Inoculum concentration (10^6 CFU/mL)**	**TSS content (°Brix)**	**SOD (U/g)**	**GTs (mg/g)**
1	20	37	5	4	108.22	3.82
2	28	37	5	5	120.57	4.19
3	20	37	2	4.5	110.39	3.93
4	28	37	2	4.5	119.13	4.14
5	24	37	5	4.5	126.81	4.41
6	20	37	5	5	115.51	4.12
7	24	32	2	4.5	97.12	3.13
8	24	37	8	4	124.36	4.31
9	24	37	5	4.5	126.02	4.33
10	24	42	8	4.5	110.47	3.85
11	24	37	5	4.5	127.85	4.39
12	20	37	8	4.5	114.63	4.01
13	24	42	5	4	108.52	3.77
14	24	37	2	4	115.25	3.97
15	28	37	5	4	116.74	4.12
16	24	32	5	4	95.58	3.31
17	24	42	2	4.5	110.29	3.65
18	24	32	5	5	100.33	3.44
19	24	37	2	5	119.38	4.03
20	28	42	5	4.5	109.14	3.79
21	20	42	5	4.5	103.20	3.71
22	24	37	8	5	126.98	4.47
23	28	32	5	4.5	95.69	3.39

24	24	32	8	4.5	99.17	3.42
25	20	32	5	4.5	85.59	3.26
26	24	37	5	4.5	126.32	4.46
27	24	42	5	5	110.97	3.42
28	24	37	5	4.5	127.86	4.39
29	28	37	8	4.5	120.44	4.19

Table S6. Analysis of variance of SOD activity and GTs in GFJ.

	SOD (U/g)			GTs (mg/g)		
**Source**	**F-Value**	***p*-Value**	**significance**	**F-Value**	***p*-Value**	**significance**
Model	82.26	<0.0001	**	22.89	<0.0001	**
A	51.80	<0.0001	**	5.82	0.0301	*
B-	166.16	<0.0001	**	31.04	<0.0001	**
C-	15.92	0.0013	**	12.12	0.0037	**
D	16.69	0.0011	**	0.85	0.3730	
AB	1.38	0.2600		0.046	0.8326	
AC	0.68	0.4222		0.017	0.8990	
AD	0.95	0.3454		0.98	0.3386	
BC	0.28	0.6059		0.15	0.7041	
BD	0.42	0.5268		4.28	0.0577	
CD	0.18	0.6765		0.19	0.6732	
A^2	148.70	<0.0001	**	14.62	0.0019	**
B^2	837.44	<0.0001	**	264.01	<0.0001	**
C^2	12.28	0.0035	**	6.99	0.0193	*
D^2	19.70	0.0006	**	9.00	0.0095	**
Lack of Fit	5.63	0.0552	Not significant	8.25	0.2838	Not significant
R^2	0.9880			0.9581		
Adj. R^2	0.9760			0.9163		
C.V. %	1.40			1.35		

Note: A: fermentation time (h), B: fermentation temperature (°C), C: inoculum

concentration, and D: soluble solid content (°Brix). * significant at 95% confidence interval. ** significant at 99% confidence interval.

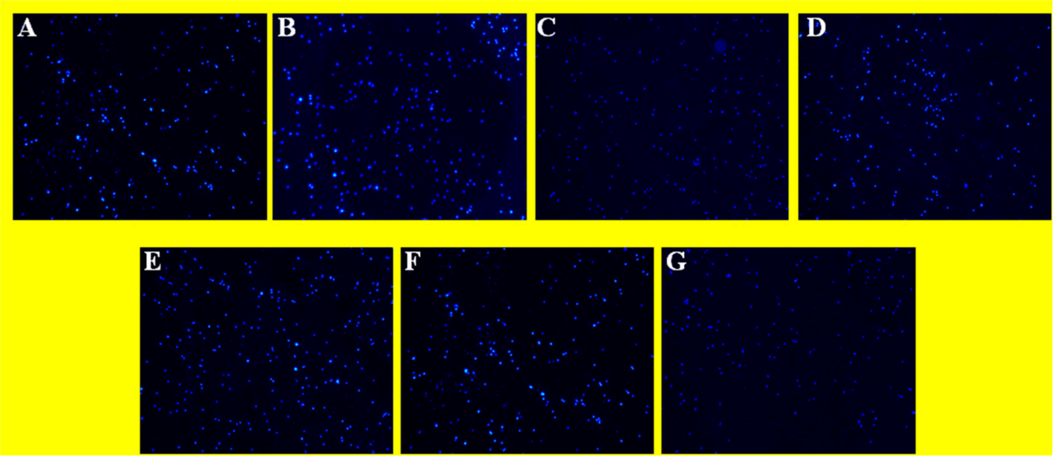

Figure S1： fluorescence inverted microscope observation: A: control group; B: LPS group; C: 0.1 mL/mL; D: 0.2 mL/mL; E: 0.3 mL/mL; F: 0.4 mL/mL; G: 0.5 mL/mL.
